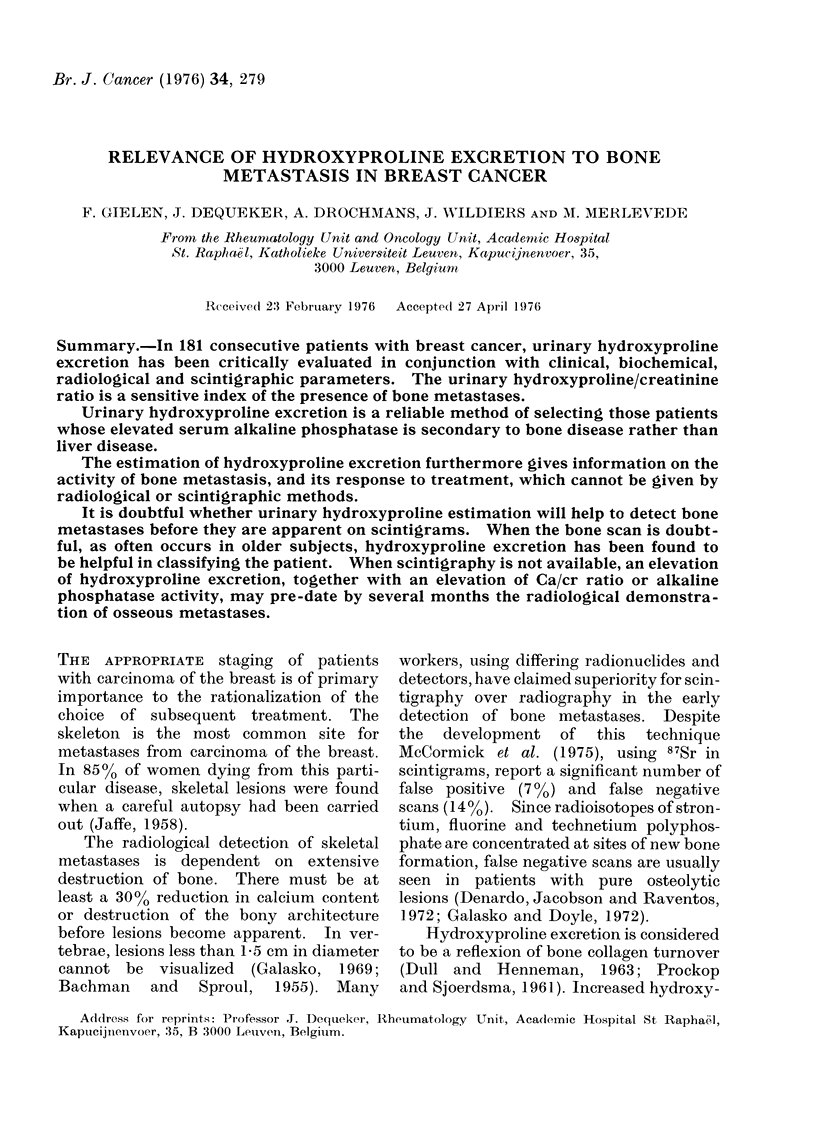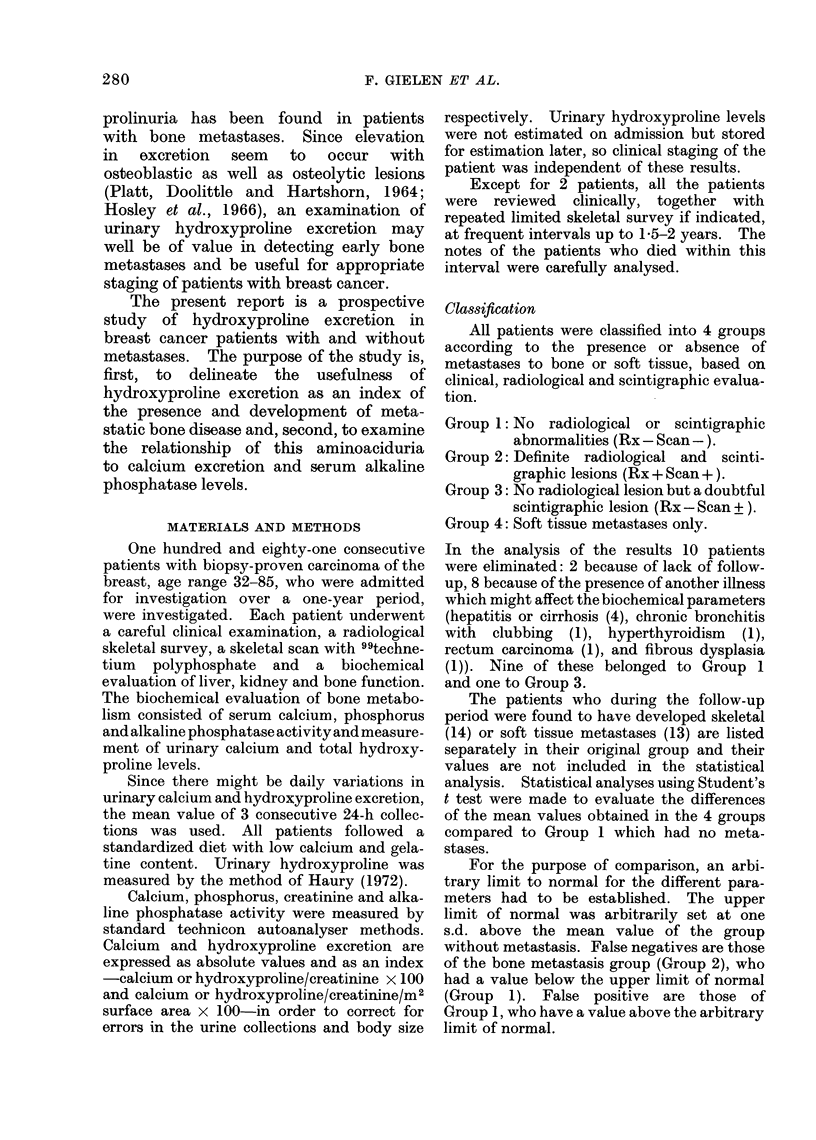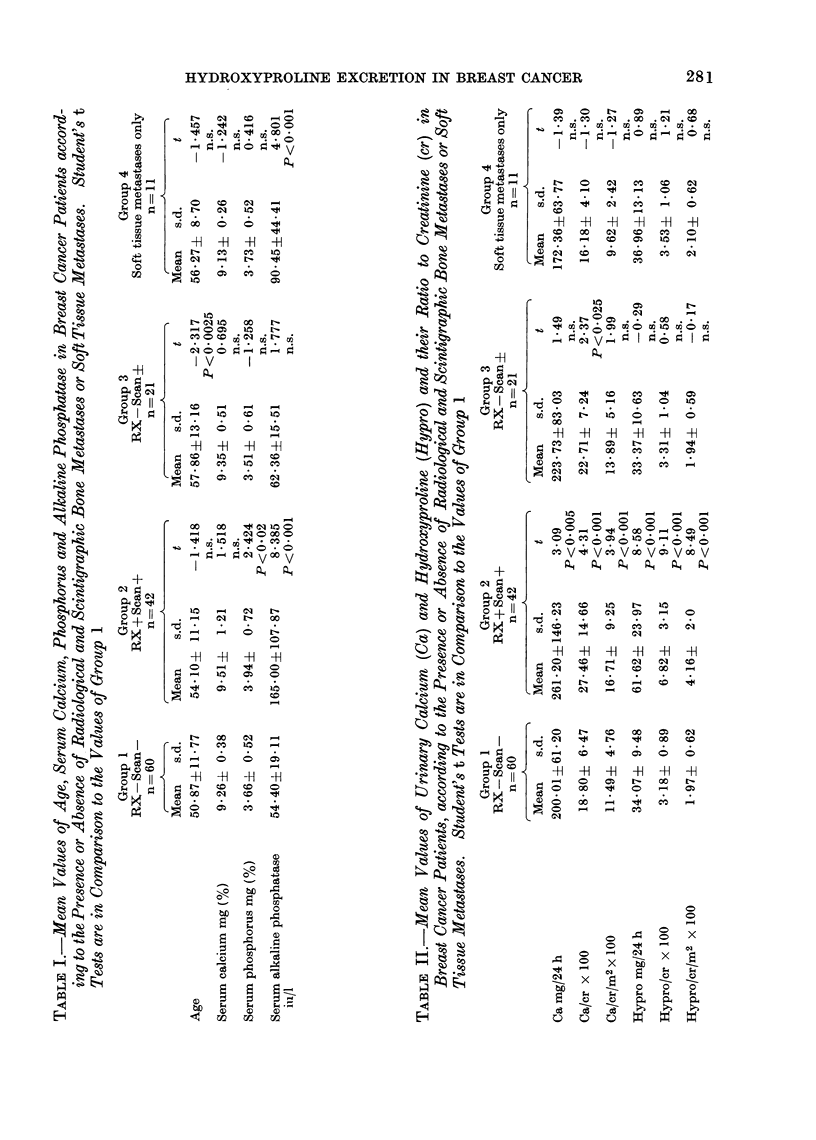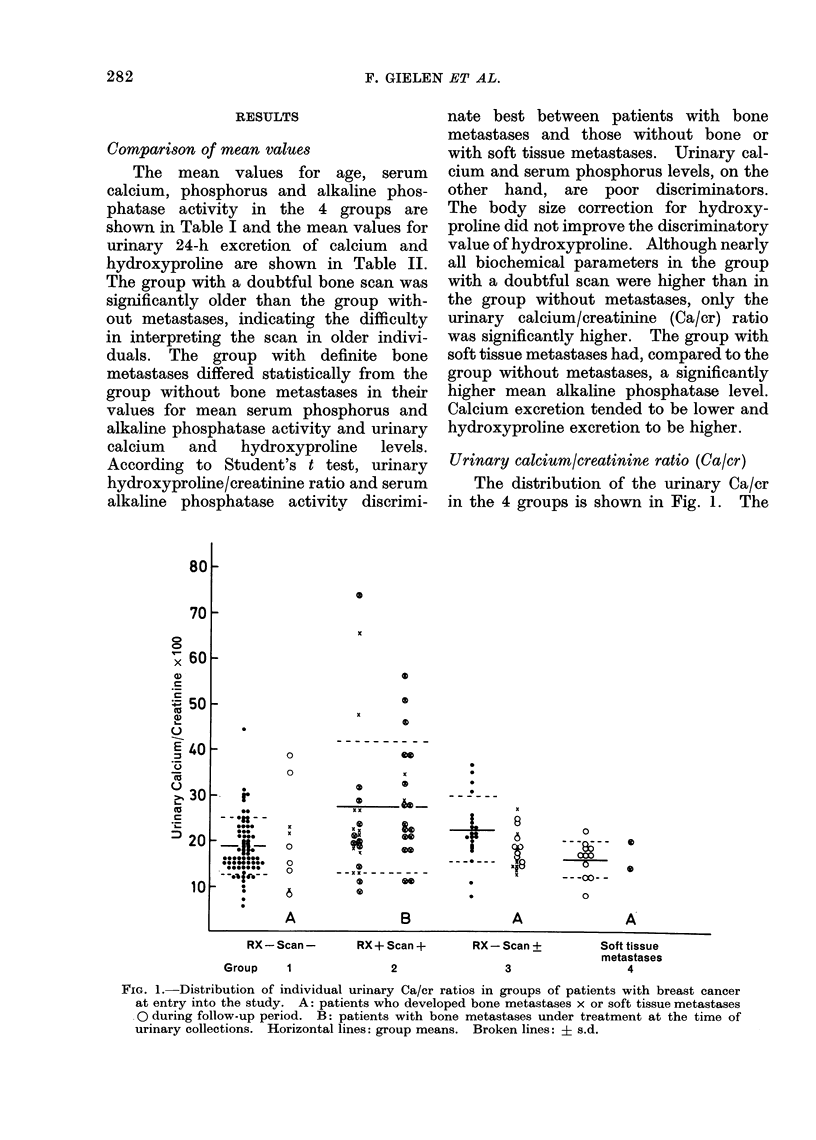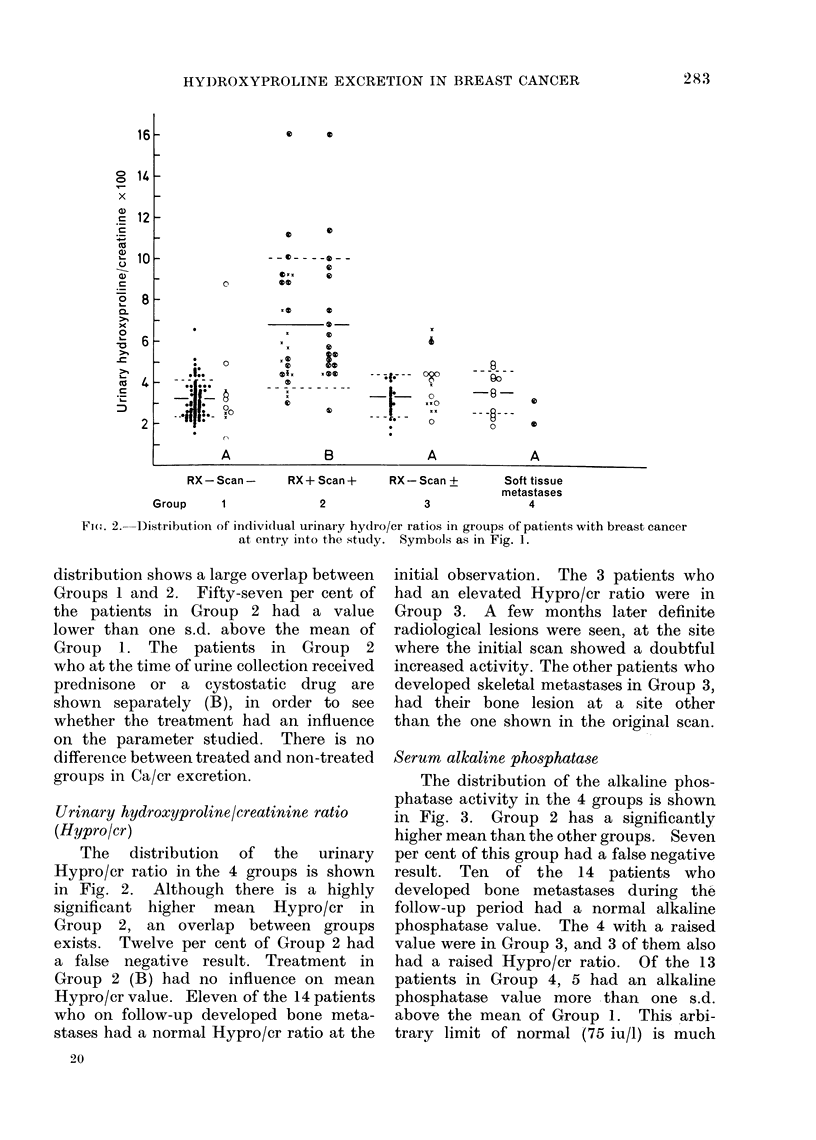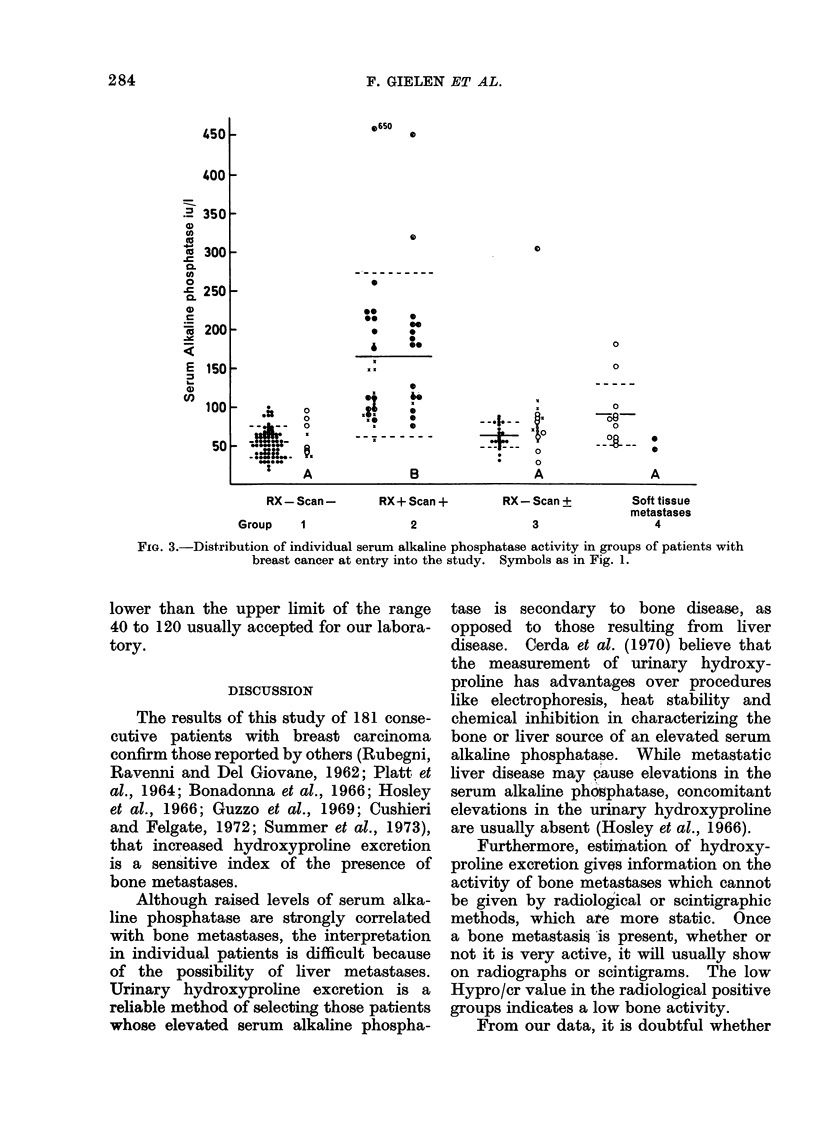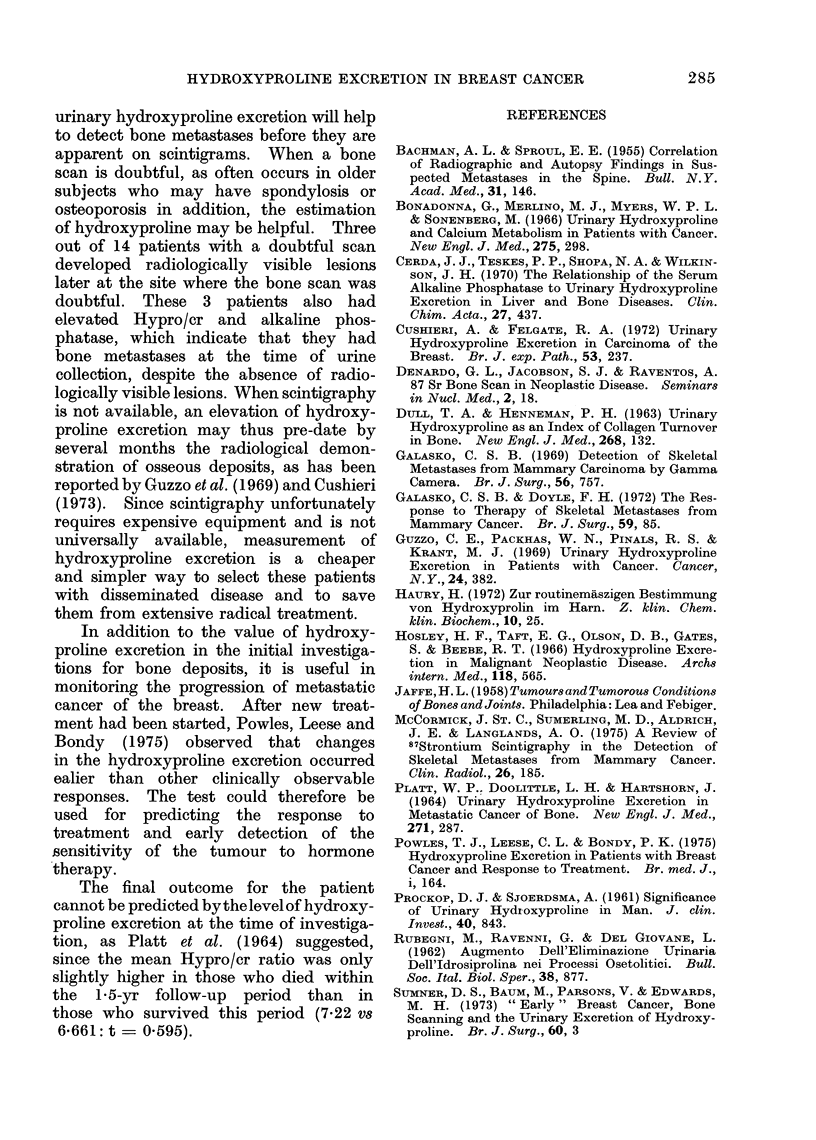# Relevance of hydroxyproline excretion to bone metastasis in breast cancer.

**DOI:** 10.1038/bjc.1976.163

**Published:** 1976-09

**Authors:** F. Gielen, J. Dequeker, A. Drochmans, J. Wildiers, M. Merlevede

## Abstract

In 181 consecutive patients with breast cancer, urinary hydroxyproline excretion has been critically evaluated in conjunction with clinical, biochemical, radiological and scintigraphic parameters. The urinary hydroxyproline/creatinine ratio is a sensitive index of the presence of bone metastases. Urinary hydroxyproline excretion is a reliable method of selecting those patients whose elevated serum alkaline phosphatase is secondary to bone disease rather than liver idsease. The estimation of hydroxyproline excretion furthermore gives information on the activity of bone metastasis, and its response to treatment, which cannot be given by radiological or scintigraphic methods. It is doubtful whether urinary hydroxyproline estimation will help to detect bone metastases before they are apparent on scintigrams. When the bone scan is doubtful, as often occurs in older subjects, hydroxyproline excretion has been found to be helpful in classifying the patient. When scintigraphy is not available, an elevation of hydroxyproline excretion, together with an elevation of Ca/cr ratio or alkaline phosphatase activity, may pre-date by several months the radiological demonstration of osseous metastases.


*Br. J. Cancer* (1976) **34**, 279

### RELEVANCE OF HYDROXYPROLINE EXCRETION TO BONE METASTASIS IN BREAST CANCER

F. GIELEN, J. DEQUEKER, A. DROCHMANS, J. WILDIERS AND M. MERLEVEDE

*From the Rheumatology Unit and Oncology Unit, Academic Hospital St. Raphaël, Katholieke Universiteit Leuven, Kapucijnenvoer, 35, 3000 Leuven, Belgium*

Received 23 February 1976 Accepted 27 April 1976

**Summary.—In 181 consecutive patients with breast cancer, urinary hydroxyproline excretion has been critically evaluated in conjunction with clinical, biochemical, radiological and scintigraphic parameters. The urinary hydroxyproline/creatinine ratio is a sensitive index of the presence of bone metastases.**

**Urinary hydroxyproline excretion is a reliable method of selecting those patients whose elevated serum alkaline phosphatase is secondary to bone disease rather than liver disease.**

**The estimation of hydroxyproline excretion furthermore gives information on the activity of bone metastasis, and its response to treatment, which cannot be given by radiological or scintigraphic methods.**

**It is doubtful whether urinary hydroxyproline estimation will help to detect bone metastases before they are apparent on scintigrams. When the bone scan is doubtful, as often occurs in older subjects, hydroxyproline excretion has been found to be helpful in classifying the patient. When scintigraphy is not available, an elevation of hydroxyproline excretion, together with an elevation of Ca/cr ratio or alkaline phosphatase activity, may pre-date by several months the radiological demonstration of osseous metastases.**

THE APPROPRIATE staging of patients with carcinoma of the breast is of primary importance to the rationalization of the choice of subsequent treatment. The skeleton is the most common site for metastases from carcinoma of the breast. In 85% of women dying from this particular disease, skeletal lesions were found when a careful autopsy had been carried out (Jaffe, 1958).

The radiological detection of skeletal metastases is dependent on extensive destruction of bone. There must be at least a 30% reduction in calcium content or destruction of the bony architecture before lesions become apparent. In vertebrae, lesions less than 1·5 cm in diameter cannot be visualized (Galasko, 1969; Bachman and Sproul, 1955). Many workers, using differing radionuclides and detectors, have claimed superiority for scintigraphy over radiography in the early detection of bone metastases. Despite the development of this technique McCormick *et al.* (1975), using $^{87}Sr$ in scintigrams, report a significant number of false positive (7%) and false negative scans (14%). Since radioisotopes of strontium, fluorine and technetium polyphosphate are concentrated at sites of new bone formation, false negative scans are usually seen in patients with pure osteolytic lesions (Denardo, Jacobson and Raventos, 1972; Galasko and Doyle, 1972).

Hydroxyproline excretion is considered to be a reflexion of bone collagen turnover (Dull and Henneman, 1963; Prockop and Sjoerdsma, 1961). Increased hydroxy-

Address for reprints: Professor J. Dequeker, Rheumatology Unit, Academic Hospital St Raphaël, Kapucijnenvoer, 35, B 3000 Leuven, Belgium.

prolinuria has been found in patients with bone metastases. Since elevation in excretion seem to occur with osteoblastic as well as osteolytic lesions (Platt, Doolittle and Hartshorn, 1964; Hosley *et al.*, 1966), an examination of urinary hydroxyproline excretion may well be of value in detecting early bone metastases and be useful for appropriate staging of patients with breast cancer.

The present report is a prospective study of hydroxyproline excretion in breast cancer patients with and without metastases. The purpose of the study is, first, to delineate the usefulness of hydroxyproline excretion as an index of the presence and development of metastatic bone disease and, second, to examine the relationship of this aminoaciduria to calcium excretion and serum alkaline phosphatase levels.

### MATERIALS AND METHODS

One hundred and eighty-one consecutive patients with biopsy-proven carcinoma of the breast, age range 32–85, who were admitted for investigation over a one-year period, were investigated. Each patient underwent a careful clinical examination, a radiological skeletal survey, a skeletal scan with $^{99}$technetium polyphosphate and a biochemical evaluation of liver, kidney and bone function. The biochemical evaluation of bone metabolism consisted of serum calcium, phosphorus and alkaline phosphatase activity and measurement of urinary calcium and total hydroxyproline levels.

Since there might be daily variations in urinary calcium and hydroxyproline excretion, the mean value of 3 consecutive 24-h collections was used. All patients followed a standardized diet with low calcium and gelatine content. Urinary hydroxyproline was measured by the method of Haury (1972).

Calcium, phosphorus, creatinine and alkaline phosphatase activity were measured by standard technicon autoanalyser methods. Calcium and hydroxyproline excretion are expressed as absolute values and as an index —calcium or hydroxyproline/creatinine $\times$ 100 and calcium or hydroxyproline/creatinine/m$^2$ surface area $\times$ 100—in order to correct for errors in the urine collections and body size respectively. Urinary hydroxyproline levels were not estimated on admission but stored for estimation later, so clinical staging of the patient was independent of these results.

Except for 2 patients, all the patients were reviewed clinically, together with repeated limited skeletal survey if indicated, at frequent intervals up to 1·5–2 years. The notes of the patients who died within this interval were carefully analysed.

### *Classification*

All patients were classified into 4 groups according to the presence or absence of metastases to bone or soft tissue, based on clinical, radiological and scintigraphic evaluation.

Group 1: No radiological or scintigraphic abnormalities (Rx − Scan −).
Group 2: Definite radiological and scintigraphic lesions (Rx + Scan +).
Group 3: No radiological lesion but a doubtful scintigraphic lesion (Rx − Scan ±).
Group 4: Soft tissue metastases only.

In the analysis of the results 10 patients were eliminated: 2 because of lack of follow-up, 8 because of the presence of another illness which might affect the biochemical parameters (hepatitis or cirrhosis (4), chronic bronchitis with clubbing (1), hyperthyroidism (1), rectum carcinoma (1), and fibrous dysplasia (1)). Nine of these belonged to Group 1 and one to Group 3.

The patients who during the follow-up period were found to have developed skeletal (14) or soft tissue metastases (13) are listed separately in their original group and their values are not included in the statistical analysis. Statistical analyses using Student's *t* test were made to evaluate the differences of the mean values obtained in the 4 groups compared to Group 1 which had no metastases.

For the purpose of comparison, an arbitrary limit to normal for the different parameters had to be established. The upper limit of normal was arbitrarily set at one s.d. above the mean value of the group without metastasis. False negatives are those of the bone metastasis group (Group 2), who had a value below the upper limit of normal (Group 1). False positive are those of Group 1, who have a value above the arbitrary limit of normal.

### RESULTS

*Comparison of mean values*

The mean values for age, serum calcium, phosphorus and alkaline phosphatase activity in the 4 groups are shown in Table I and the mean values for urinary 24-h excretion of calcium and hydroxyproline are shown in Table II. The group with a doubtful bone scan was significantly older than the group without metastases, indicating the difficulty in interpreting the scan in older individuals. The group with definite bone metastases differed statistically from the group without bone metastases in their values for mean serum phosphorus and alkaline phosphatase activity and urinary calcium and hydroxyproline levels. According to Student's $t$ test, urinary hydroxyproline/creatinine ratio and serum alkaline phosphatase activity discriminate best between patients with bone metastases and those without bone or with soft tissue metastases. Urinary calcium and serum phosphorus levels, on the other hand, are poor discriminators. The body size correction for hydroxyproline did not improve the discriminatory value of hydroxyproline. Although nearly all biochemical parameters in the group with a doubtful scan were higher than in the group without metastases, only the urinary calcium/creatinine (Ca/cr) ratio was significantly higher. The group with soft tissue metastases had, compared to the group without metastases, a significantly higher mean alkaline phosphatase level. Calcium excretion tended to be lower and hydroxyproline excretion to be higher.

*Urinary calcium/creatinine ratio (Ca/cr)*

The distribution of the urinary Ca/cr in the 4 groups is shown in Fig. 1. The

FIG. 1.—Distribution of individual urinary Ca/cr ratios in groups of patients with breast cancer at entry into the study. A: patients who developed bone metastases × or soft tissue metastases ○ during follow-up period. B: patients with bone metastases under treatment at the time of urinary collections. Horizontal lines: group means. Broken lines: ± s.d.

FIG. 2.—Distribution of individual urinary hydro/cr ratios in groups of patients with breast cancer at entry into the study. Symbols as in Fig. 1.

distribution shows a large overlap between Groups 1 and 2. Fifty-seven per cent of the patients in Group 2 had a value lower than one s.d. above the mean of Group 1. The patients in Group 2 who at the time of urine collection received prednisone or a cystostatic drug are shown separately (B), in order to see whether the treatment had an influence on the parameter studied. There is no difference between treated and non-treated groups in Ca/cr excretion.

*Urinary hydroxyproline/creatinine ratio (Hypro/cr)*

The distribution of the urinary Hypro/cr ratio in the 4 groups is shown in Fig. 2. Although there is a highly significant higher mean Hypro/cr in Group 2, an overlap between groups exists. Twelve per cent of Group 2 had a false negative result. Treatment in Group 2 (B) had no influence on mean Hypro/cr value. Eleven of the 14 patients who on follow-up developed bone metastases had a normal Hypro/cr ratio at the initial observation. The 3 patients who had an elevated Hypro/cr ratio were in Group 3. A few months later definite radiological lesions were seen, at the site where the initial scan showed a doubtful increased activity. The other patients who developed skeletal metastases in Group 3, had their bone lesion at a site other than the one shown in the original scan.

*Serum alkaline phosphatase*

The distribution of the alkaline phosphatase activity in the 4 groups is shown in Fig. 3. Group 2 has a significantly higher mean than the other groups. Seven per cent of this group had a false negative result. Ten of the 14 patients who developed bone metastases during the follow-up period had a normal alkaline phosphatase value. The 4 with a raised value were in Group 3, and 3 of them also had a raised Hypro/cr ratio. Of the 13 patients in Group 4, 5 had an alkaline phosphatase value more than one s.d. above the mean of Group 1. This arbitrary limit of normal (75 iu/l) is much

FIG. 3.—Distribution of individual serum alkaline phosphatase activity in groups of patients with breast cancer at entry into the study. Symbols as in Fig. 1.

lower than the upper limit of the range 40 to 120 usually accepted for our laboratory.

### DISCUSSION

The results of this study of 181 consecutive patients with breast carcinoma confirm those reported by others (Rubegni, Ravenni and Del Giovane, 1962; Platt *et al.*, 1964; Bonadonna *et al.*, 1966; Hosley *et al.*, 1966; Guzzo *et al.*, 1969; Cushieri and Felgate, 1972; Summer *et al.*, 1973), that increased hydroxyproline excretion is a sensitive index of the presence of bone metastases.

Although raised levels of serum alkaline phosphatase are strongly correlated with bone metastases, the interpretation in individual patients is difficult because of the possibility of liver metastases. Urinary hydroxyproline excretion is a reliable method of selecting those patients whose elevated serum alkaline phosphatase is secondary to bone disease, as opposed to those resulting from liver disease. Cerda *et al.* (1970) believe that the measurement of urinary hydroxyproline has advantages over procedures like electrophoresis, heat stability and chemical inhibition in characterizing the bone or liver source of an elevated serum alkaline phosphatase. While metastatic liver disease may cause elevations in the serum alkaline phosphatase, concomitant elevations in the urinary hydroxyproline are usually absent (Hosley *et al.*, 1966).

Furthermore, estimation of hydroxyproline excretion gives information on the activity of bone metastases which cannot be given by radiological or scintigraphic methods, which are more static. Once a bone metastasis is present, whether or not it is very active, it will usually show on radiographs or scintigrams. The low Hypro/cr value in the radiological positive groups indicates a low bone activity.

From our data, it is doubtful whether urinary hydroxyproline excretion will help to detect bone metastases before they are apparent on scintigrams. When a bone scan is doubtful, as often occurs in older subjects who may have spondylosis or osteoporosis in addition, the estimation of hydroxyproline may be helpful. Three out of 14 patients with a doubtful scan developed radiologically visible lesions later at the site where the bone scan was doubtful. These 3 patients also had elevated Hypro/cr and alkaline phosphatase, which indicate that they had bone metastases at the time of urine collection, despite the absence of radiologically visible lesions. When scintigraphy is not available, an elevation of hydroxyproline excretion may thus pre-date by several months the radiological demonstration of osseous deposits, as has been reported by Guzzo *et al.* (1969) and Cushieri (1973). Since scintigraphy unfortunately requires expensive equipment and is not universally available, measurement of hydroxyproline excretion is a cheaper and simpler way to select these patients with disseminated disease and to save them from extensive radical treatment.

In addition to the value of hydroxyproline excretion in the initial investigations for bone deposits, it is useful in monitoring the progression of metastatic cancer of the breast. After new treatment had been started, Powles, Leese and Bondy (1975) observed that changes in the hydroxyproline excretion occurred ealier than other clinically observable responses. The test could therefore be used for predicting the response to treatment and early detection of the sensitivity of the tumour to hormone therapy.

The final outcome for the patient cannot be predicted by the level of hydroxyproline excretion at the time of investigation, as Platt *et al.* (1964) suggested, since the mean Hypro/cr ratio was only slightly higher in those who died within the 1·5-yr follow-up period than in those who survived this period (7·22 *vs* 6·661: $t = 0{\cdot}595$).

### REFERENCES

Bachman, A. L. & Sproul, E. E. (1955) Correlation of Radiographic and Autopsy Findings in Suspected Metastases in the Spine. *Bull. N.Y. Acad. Med.*, **31**, 146.

Bonadonna, G., Merlino, M. J., Myers, W. P. L. & Sonenberg, M. (1966) Urinary Hydroxyproline and Calcium Metabolism in Patients with Cancer. *New Engl. J. Med.*, **275**, 298.

Cerda, J. J., Teskes, P. P., Shopa, N. A. & Wilkinson, J. H. (1970) The Relationship of the Serum Alkaline Phosphatase to Urinary Hydroxyproline Excretion in Liver and Bone Diseases. *Clin. Chim. Acta.*, **27**, 437.

Cushieri, A. & Felgate, R. A. (1972) Urinary Hydroxyproline Excretion in Carcinoma of the Breast. *Br. J. exp. Path.*, **53**, 237.

Denardo, G. L., Jacobson, S. J. & Raventos, A. 87 Sr Bone Scan in Neoplastic Disease. *Seminars in Nucl. Med.*, **2**, 18.

Dull, T. A. & Henneman, P. H. (1963) Urinary Hydroxyproline as an Index of Collagen Turnover in Bone. *New Engl. J. Med.*, **268**, 132.

Galasko, C. S. B. (1969) Detection of Skeletal Metastases from Mammary Carcinoma by Gamma Camera. *Br. J. Surg.*, **56**, 757.

Galasko, C. S. B. & Doyle, F. H. (1972) The Response to Therapy of Skeletal Metastases from Mammary Cancer. *Br. J. Surg.*, **59**, 85.

Guzzo, C. E., Packhas, W. N., Pinals, R. S. & Krant, M. J. (1969) Urinary Hydroxyproline Excretion in Patients with Cancer. *Cancer, N.Y.*, **24**, 382.

Haury, H. (1972) Zur routinemäszigen Bestimmung von Hydroxyprolin im Harn. *Z. klin. Chem. klin. Biochem.*, **10**, 25.

Hosley, H. F., Taft, E. G., Olson, D. B., Gates, S. & Beebe, R. T. (1966) Hydroxyproline Excretion in Malignant Neoplastic Disease. *Archs intern. Med.*, **118**, 565.

Jaffe, H. L. (1958) *Tumours and Tumorous Conditions of Bones and Joints*. Philadelphia: Lea and Febiger.

McCormick, J. St. C., Sumerling, M. D., Aldrich, J. E. & Langlands, A. O. (1975) A Review of $^{87}$Strontium Scintigraphy in the Detection of Skeletal Metastases from Mammary Cancer. *Clin. Radiol.*, **26**, 185.

Platt, W. P., Doolittle, L. H. & Hartshorn, J. (1964) Urinary Hydroxyproline Excretion in Metastatic Cancer of Bone. *New Engl. J. Med.*, **271**, 287.

Powles, T. J., Leese, C. L. & Bondy, P. K. (1975) Hydroxyproline Excretion in Patients with Breast Cancer and Response to Treatment. *Br. med. J.*, i, 164.

Prockop, D. J. & Sjoerdsma, A. (1961) Significance of Urinary Hydroxyproline in Man. *J. clin. Invest.*, **40**, 843.

Rubegni, M., Ravenni, G. & Del Giovane, L. (1962) Augmento Dell'Eliminazione Urinaria Dell'Idrosiprolina nei Processi Osetolitici. *Bull. Soc. Ital. Biol. Sper.*, **38**, 877.

Sumner, D. S., Baum, M., Parsons, V. & Edwards, M. H. (1973) "Early" Breast Cancer, Bone Scanning and the Urinary Excretion of Hydroxyproline. *Br. J. Surg.*, **60**, 3